# Successful Treatment of Anterior Tracheal Necrosis after Total Thyroidectomy Using Vacuum-Assisted Closure Therapy

**DOI:** 10.1155/2012/252719

**Published:** 2011-06-20

**Authors:** Grégory Philippe, Nicolas Pichon, Justine Lerat, Jean Bernard Amiel, Marc Clavel, Muriel Mathonnet

**Affiliations:** ^1^Department of Anesthesiology, Dupuytren University Hospital, 87042 Limoges, France; ^2^Department of Critical Care, Dupuytren University Hospital, 87042 Limoges, France; ^3^Department of Ear, Nose and Throat Surgery, Dupuytren University Hospital, 87042 Limoges, France; ^4^Department of Surgery, Dupuytren University Hospital, 87042 Limoges, France

## Abstract

Total thyroidectomy involving the adjacent structures of the trachea can cause tracheal damage such as early tracheal necrosis. The authors describe the first case of anterior tracheal necrosis following total thyroidectomy treated using vacuum-assisted closure device. After two weeks of VAC  therapy, there was no evidence of ongoing infection and the trachea was partially closed around a tracheotomy cannula, removed after 3 months. The use of a VAC  therapy to reduce and close the tracheal rent and to create a rapid granulation tissue over tracheal structure appeared as a good opportunity after anterior tracheal necrosis.

## 1. Introduction

Surgery involving the adjacent structures of the trachea can cause tracheal damage such as neck abscess and tracheal necrosis [1]. Early anterior tracheal necrosis following total thyroidectomy has been reported only twice [2, 3]. How to treat such a complication is not standardized. The authors describe the first case of postoperative necrosis of the upper anterior part of the cervical trachea following total thyroidectomy, treated using systemic antibiotherapy and vacuum-assisted closure (VAC) Therapy. 

## 2. Case Report

**Figure 1:**
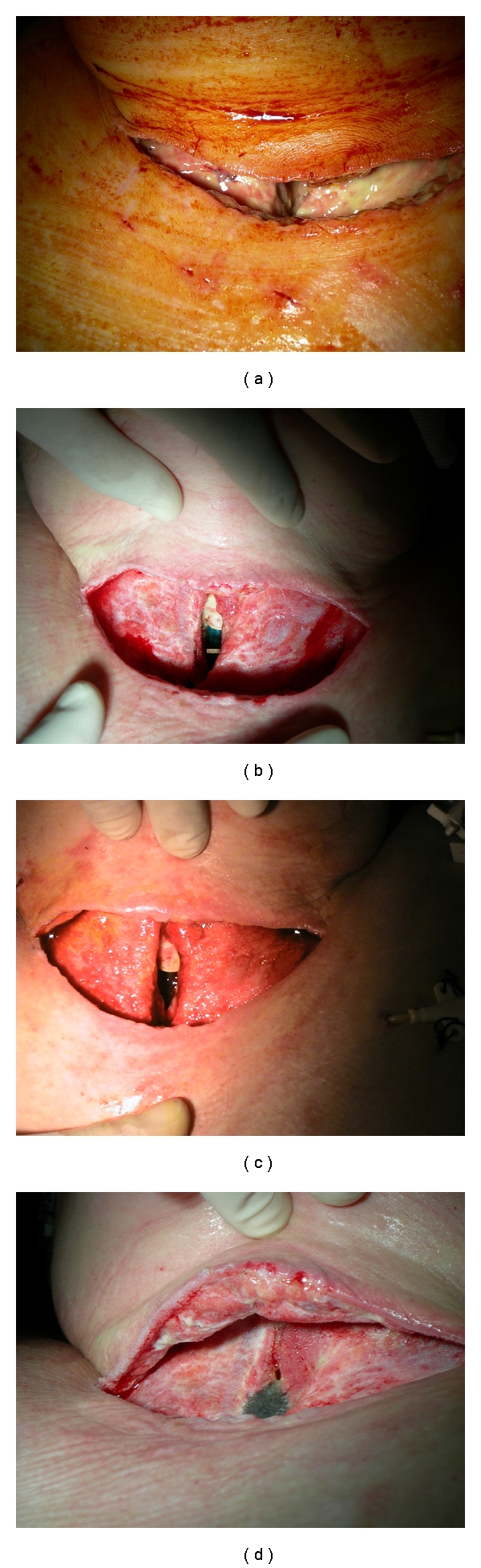
Time course of anterior tracheal necrosis under treatment.

**Figure 2:**
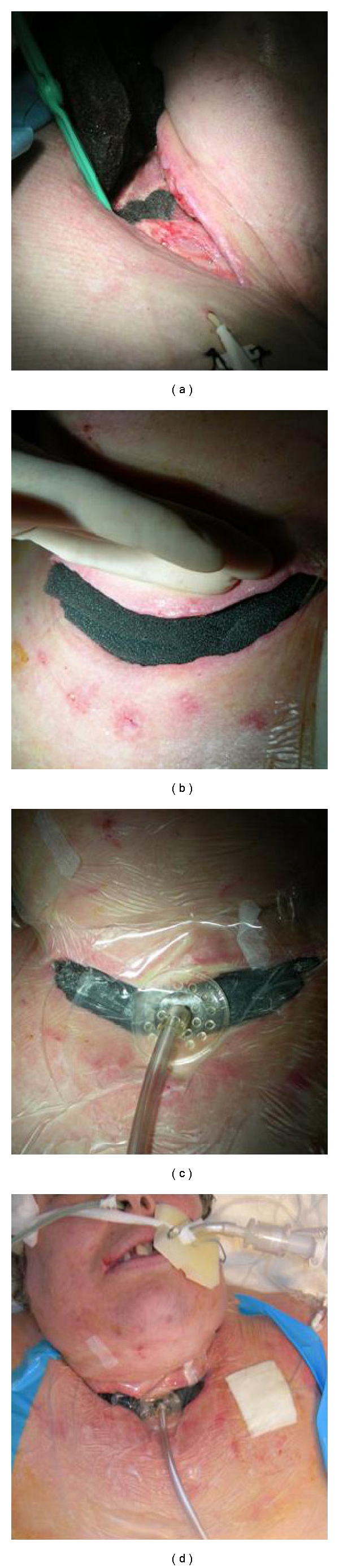
Vacuum-assisted closure therapy.

A 56-year-old woman, with a past medical and surgical history significant for obesity (body mass index: 43), arterial hypertension, hysterectomy 15 years ago, and ovarian adenocarcinoma treated by ovariectomy and adjuvant chemotherapy 1 year ago, underwent elective uneventful total thyroidectomy, using cautery, and bilateal upper parathyroidectomy preserving the recurrent laryngeal nerves and inferior parathyroid glands by an experienced senior surgeon under general anesthesia. Thyroid vesicular adenocarcinoma (pT1N0M0) was confirmed after histological analysis. Tracheal intubation using a 7 mm tracheal tube was easy, and tracheal cuff was inflated with air until no leak was detected. Postoperatively, she was weaned from mechanical ventilation but an acute respiratory distress immediately appeared, requiring immediate endotracheal intubation of the patient's trachea and consecutive mechanical ventilation. A left recurrent laryngeal nerve paralysis was diagnosed and confirmed by a postoperative ENT check. The patient was admitted into intensive care unit, and a systemic corticotherapy (1 mg/kg/day) was introduced. A new attempt of ventilatory weaning was performed 4 days later with a second acute respiratory distress and a new endotracheal intubation. On the sixth postoperative day, the patient presented a productive cough, a temperature of 38.2°C, and a suspicion of wound infection of the operative site and developed rapidly an increasing emphysema of the neck and the upper torso. Computerized tomodensitometric cervical and thoracic examination showed a pneumomediastinum and a leak from the anterior part of the trachea secondary to localized tracheal necrosis confirmed by fiberoptic bronchoscopy. Prompt surgical exploration of the neck revealed a necrotic anterior part of the trachea involving the second to the fifth tracheal rings and a perforation of the third and fourth rings (Figure 1(a)). The lateral parts and the posterior membranous part of the trachea were intact. The necrosis of the tracheal wall was not located in the area of the cuff extending. The necrotic trachea was excised, leaving a large anterior defect that spanned 4 rings (Figure 1(b)). There was an inability for surgeons to perform a sleeve resection with primary anastomosis, a muscle flap reconstruction of the anterior trachea, or a partial tracheal closing around a Montgomery T-tube or tracheotomy canula because there was a too high risk causing the acute septic process to spread and because the patient presented both life-threatening cardiovascular and respiratory failures. The endotracheal tube was left long, and the cuff was advanced beyond the tracheal necrotic area. At the time of the first aggressive tracheal and cervical wound debridement, a vacuum-assisted closure device (VAC therapy) was used to manage the wound (Figure 2). A polyurethane sponge cell foam dressing ((VAC GranuFoam), recommended for stimulating granulation tissue and wound contraction, was cut to be slightly smaller than the wound (Figure 2(a)), placed directly onto the wound (Figure 2(b)), and loosely covered with a clear occlusive drape to encompass the entire wound (Figure 2(c)). A separate small piece of foam was place in the defect to keep a hole to be refashioned into a formal tracheostomy allowing the later insertion of a tracheotomy canula (Figures 1(d) and 2(a)). Placing the drape loosely allowed the suction to create a tight seal along the perimeter of the wound and decreased periwound maceration. The suction tubing was placed into the sponge at the end of the application of the dressing (Figure 2(c)) and covered with an additional piece of OpSite (Smith & Nephew), making sure to pinch the drape around the tubing to create a complete seal (Figure 2(d)). The occlusive dressing was applied lightly over the sponge and wound, trying to lay the adhesive dressing down like a blanket instead of a tent. This helps to reduce any free air spaces which can make a complete seal difficult. This step over irregular surfaces such as the neck often requires multiple sheets of dressing. Using a scalpel, the occlusive dressing and the outer portions of the sponge were incised to facilitate insertion of the VAC line. The dressing was then connected to a vacuum pump (VAC ATS), providing a continuous subatmospheric pressure setting to 75 mm Hg. Bacterial examination of the cervical damaged area revealed strains of *Escherichia coli*, *Streptococcus C*, and *Staphylococcus aureus*. Antibiotics were administered for 3 weeks. The dressing change interval at our center was 72 hours and was performed in 15 to 20 minutes. Each time, the dressing was changed at bedside and the wound was debrided to decrease the amount of fibrotic and nonviable tissue (Figure 1(c)). The surrounding tissue was cleaned and completely dry until granulation tissue partially covered tracheal structures (Figure 1(d)). There was no complication associated with utilization of VAC. After two weeks of VAC therapy, 3 debridements, and dressing changes, there was no evidence of ongoing infection and the trachea was partially closed around a no. 7 tracheotomy canula. The use of the VAC therapy improved the soft tissue component of the neck wound and likely impacted the tracheal wound. The patient was discharged with the canula in place for 3 months. An endoscopic examination was then performed showing a complete closure of the trachea around the tracheotomy canula, no sign of local infection, tracheal stenosis or malacia, and no more left recurrent laryngeal nerve paralysis. The tracheotomy canula was then removed. At 2 years, the patient is well with no tracheal symptoms. There is no immobilization of the trachea but the resultant cervical skin scar-tissue adheres to the trachea over two inches in height.

## 3. Discussion

Several factors are implicated in tracheal damage after intubation and thyroidectomy including prolonged intubation, inadequate tube size, traumatic maneuvers during tube insertion or mobilization of a tracheal tube prior to sufficient tube cuff deflation, hyperinflation of the cuff exceeding the perfusion pressure of the tracheal mucosa (low perfusion pressure of 25 mm Hg has been recorded in tracheal mucosal capillaries) and resulting in subsequent membranous part ischemia and tissue necrosis, use of excessive cautery on and around the trachea, disruption of blood supply to the trachea, postoperative development of a hematoma or an untoward intraoperative tracheal perforation creating an environment for bacteria to seed and resulting in localized infection and necrosis [2–7]. Our patient had a number of predisposing risk factors to develop tracheal injury and subsequent secondary infection leading to tracheal necrosis. First, excessive pressure from the cuff (there was no monitoring of the pressure in the cuff during and after thyroidectomy in our case) or perhaps trauma from the tip of the endotracheal tube during one of the three endotracheal intubations performed may have caused injury to the trachea. Second, we suspect that excessive cauterization was employed for hemostasis and compromised trachea blood supply. Third, the development of a cervical hematoma and the damaged tracheal wall created an environment for bacteria to seed, resulting in infection and necrosis. Finally, the introduction of a systemic corticotherapy, to treat the left recurrent laryngeal nerve paralysis, exposed the patient to infection of the cervical hematoma. One of the major complications directly attributed to total thyroidectomy is recurrent laryngeal nerve injury, particularly if such injury involves the nerve contralateral to a primary thyroid tumor. The reported incidence of transient recurrent laryngeal nerve injury after thyroidectomy is 6.2%. The occurrence of recurrent laryngeal nerve injury is attributed to operative technique, extent of the thyroid neoplasm, concomitant lymphatic dissections, and other factors such as anatomic variability and vulnerability of the recurrent laryngeal nerves moreover when electrocoagulation is used for surgery. It is known that excessive cautery done to control bleeding during thyroidectomy has the potential risk of injuring the surrounding structures from lateral dispersion of heat [5]. 

For many years, chronic and acute wounds have been the subject of intense research in an effort to find methods to increase healing rates and decrease complications. Manipulation of the macroscopic and microscopic environments of wounds has been the key to success in healing both the acute and chronic wounds. Wound treatments have ranged from the simple but effective wet-to-dry dressing to topical and systemic pharmacotherapy and biologic agents, including growth factors and skin substitutes or grafts. Since 1995, several studies have looked at the efficacy of the wound VAC therapy in increasing granulation tissue and decreasing mean time to wound closure in various types of wounds [8]. Understanding the usefulness of VAC therapy requires knowledge of the basic science behind a chronic or an acute wound. Wounds typically represent a breakdown in the transition between the substrate and proliferative stages of wound healing. Many factors, such as vascular disease, diabetes, pressure, infection, environmental stress, age, nutrition, immune status, and pharmacologic agents (both systemic and topical), have been reported to affect the wound environment adversely. Using the subatmospheric pressure of VAC therapy can alter the wound environment by reducing bacterial load and chronic, often inflammatory, interstitial wound exudates, potentially increasing vascularity and cytokine expression, and physically contracting wound margins. All of these characteristics, particularly the removal of deleterious proteases, may help to convert a necrotic wound into a red carpet of healthy granulation tissue so it may progress through the subsequent phases of wound healing. In our case, VAC therapy provided an additional benefit that conventional surgical dressing changes would not have offered. After two weeks of VAC therapy, the authors estimated there was no benefit to perform a consolidation treatment with flap coverage of the cervical wound.

## 4. Conclusion

As in every wound except for the most ischemic, surgical debriding is essential. A thorough lavage/cleaning of the wound is important to reduce overall bacterial colonization and to further remove nonviable matter. In our case, the tracheal necrosis was too impaired and large for a resection with primary anastomosis or partial tracheal closing around a tracheotomy canula. The use of a VAC therapy to reduce and close the tracheal rent and to create a rapid granulation tissue over tracheal structure appeared as a good opportunity. This modality is beneficial for its ability to increase local vascularity and decrease chronic inflammatory exudates and nonviable tissue. The VAC therapy must be discontinued after a significant improvement in the wound size and depth.
